# Levels of enzyme activities in six lysosomal storage diseases in Japanese neonates determined by liquid chromatography-tandem mass spectrometry

**DOI:** 10.1016/j.ymgmr.2016.08.007

**Published:** 2016-08-31

**Authors:** Ryuichi Mashima, Eri Sakai, Motomichi Kosuga, Torayuki Okuyama

**Affiliations:** a Department of Clinical Laboratory Medicine, National Center for Child Health and Development, 2-10-1 Okura, Setagaya-ku, Tokyo 157-8535, Japan; b Center for Lysosomal Storage Disorders, National Center for Child Health and Development, 2-10-1 Okura, Setagaya-ku, Tokyo 157-8535, Japan; c Division of Medical Genetics, National Center for Child Health and Development, 2-10-1 Okura, Setagaya-ku, Tokyo 157-8535, Japan

## Abstract

Lysosomal storage disorders (LSDs) are caused by defective enzyme activities in lysosomes, characterized by the accumulation of glycolipids, oligosaccharides, mucopolysaccharides, sphingolipids, and other biological substances. Accumulating evidence has suggested that early detection of individuals with LSDs, followed by the immediate initiation of appropriate therapy during the presymptomatic period, usually results in better therapeutic outcomes. The activities of individual enzymes are measured using fluorescent substrates. However, the simultaneous determination of multiple enzyme activities has been awaited in neonatal screening of LSDs because the prevalence of individual LSDs is rare. In this study, the activities of six enzymes associated with LSDs were examined with 6-plex enzyme assay using liquid chromatography-tandem mass spectrometry (LC-MS/MS). The accumulation of enzyme products was almost linear for 0–20 h at 37 °C. Dried blood spots (DBSs) provided by the Centers for Disease Control and Prevention (CDC) were used for quality control (QC). The intraday and interday coefficient of variance values were < 25%. The enzyme activities of healthy individuals were higher than those of LSD-confirmed individuals. These results suggest that the levels of enzyme activities of six LSDs in a Japanese population were comparable to those of a recent report [Elliott et al. Mol Genet Metab 118 (2016) 304–309], providing additional evidence that the 6-plex LSD enzyme assay is a reproducible analytical procedure for neonatal screening.

## 1 Introduction

Lysosomal storage disorders (LSDs) are a group of congenital metabolic disorders caused by the accumulation of glycolipids, oligosaccharides, mucopolysaccharides, sphingolipids, and other biological substances induced by the defective activity of lysosomal enzymes [1], [2]. Lysosomal enzymes are found in nearly all mammalian cells. Thus, lysosomal enzyme deficiency can lead to systemic manifestations of LSD symptoms. As regards therapy, a prior study demonstrated the efficacy of hematopoietic stem cell transplantation [3]. In this therapy, the wild-type enzyme in the transplanted hematopoietic cells is delivered to affected cells via a mechanism called cross-correction [4]. Enzyme replacement therapy plays an important role in the treatment of several LSDs, such as Pompe, Fabry, and Gaucher disease and mucopolysaccharidosis (MPS) I, II, and VI [5]. Substrate reduction therapy is also used to treat Gaucher disease [6].

Neonatal screening of six LSDs involving Pompe, Fabry, MPS I, Gaucher, Krabbe, and Niemann–Pick disease type A/B has attracted much attention [7]. Pompe disease is associated with defective α-glucosidase (GAA) activity, leading to muscle weakness [8]. Fabry disease is an X-linked disorder caused by a deficiency of α-galactosidase A (GLA), resulting in the accumulation of glycosphingolipids in biological fluids and tissues [9]. MPS I is linked to a deficiency of α-l-iduronidase (IDUA) [9]. There are three disease subtypes based on the phenotype: Hurler (severe), Hurler–Scheie (intermediate), and Scheie (mild) [10]. Gaucher disease is characterized by an accumulation of galactosylceramide due to a glucocerebrosidase (ABG) defect [11]. Krabbe disease leads to severe neurological manifestations and is linked to a galactosylceramidase (GALC) deficiency [11]. Currently, hematopoietic cell transplant therapy is used to treat Krabbe disease [3]. Niemann–Pick disease type A/B is caused by a deficiency of acid sphingomyelinase (ASM) [12]. Enzyme replacement therapy for the disease is currently under development (ClinicalTrials.gov: NCT01722526) [12].

To identify individuals with LSDs during the presymptomatic period, neonatal screening for LSDs has been performed [13], [14], [15]. In addition to neonatal screening, increasing evidence indicates that liquid chromatography-tandem mass spectrometry (LC-MS/MS)-based technology may be superior to previously performed assays for LSDs (Reviewed in [7]). Furthermore, accumulating evidence suggests that the MS/MS-based method seems to be feasible for neonatal screening [16], [17], [18], [19], [20]. In this study, we examined the ability of LC-MS/MS to detect 6-plex LSD enzyme activity and applied the methodology to random Japanese neonates.

## 2 Experimental procedure

### 2.1 Reagents

The substrates and internal standards for GAA, GLA, IDUA, ABG, ASM, and GALC were purchased from PerkinElmer (Waltham, MA). Acetonitrile and methanol were purchased from Fischer Scientific (Tokyo, Japan). Isopropanol was purchased from Wako Pure Chemicals (Tokyo, Japan). Deionized water was obtained through a Milli-Q water system from Millipore (Milford, MA). Formic acid was purchased from Kanto Chemical (Tokyo, Japan). The other reagents used in this study were of the highest grade commercially available.

### 2.2 Approval by institutional research ethics board

This study was approved by the Research Ethics Board of the National Center for Child Health and Development.

### 2.3 Dried blood spot (DBS) specimens for quality control (QC)

The DBSs for QC were kindly provided by Dr. Hui Zhou at the Newborn Screening and Molecular Biology Branch, Centers for Disease Control and Prevention (CDC) (Atlanta, GA) [21].

### 2.4 Determination of enzyme activities of six LSDs by LC-MS/MS

The preparation and analysis of the enzyme activities of the six LSDs by LC-MS/MS has been previously reported [22]. In brief, the enzymes were extracted from the DBSs (3 mm in diameter) using a punch and reacted with substrate in a buffer (30 μL) for 20 h at 37 °C in a 96-well plate. The concentrations of the substrates and internal standards were as follows: GAA, 0.35 mM, 24 μM; GLA, 1.2 mM, 24 μM; IDUA, 0.25 mM, 15 μM; ABG, 0.5 mM, 20 μM; ASM, 0.75 mM, 15 μM; and GALC, 0.85 mM, 10 μM. To terminate the reaction, a mixture of ethyl acetate/methanol (50/50, 100 μL) was added. This reaction mixture was then transferred to a 96-well deep plate, and ethyl acetate (400 μL) and water (200 μL) were added. After mixing and centrifugation, the supernatant (75 μL) was transferred to a 96-well shallow plate. This organic solution was then dried under an N_2_ stream, reconstituted with the mobile phase (150 μL, H_2_O/CH_3_CN/formic acid in a 20/80/0.002 ratio), and analyzed using an LC-MS/MS equipped with a Quattro Premier mass spectrometer and an ACQUITY ultra-high performance liquid chromatograph (Waters, Milford, MA) system. The activity of each enzyme was determined by the accumulation of the reaction product using the corresponding internal standard in μmol/h/L of blood, where each 3-mm DBS punch contained 3 μL of blood. The following analytical columns were tested: an ACQUITY BEH C18 (1.7 μm, 100 × 2.1 mm) from Waters, a Chromolith RP-2 (3 μm, 100 × 2.1 mm) from Merck-Millipore (Tokyo, Japan), and a MonoTower C18 (3 μm, 100 × 2.1 mm) from GL Sciences (Tokyo, Japan). Multiple-reaction monitoring (MRM) was used for the quantitation of enzyme reaction products. Details of the methods are available in Supplementary Tables 1–4.

### 2.5 Determination of GAA enzyme activity using a fluorometric substrate

The measurement of GAA enzyme activity was performed using 4-methylumbelliferone-labeled substrate, as reported previously [15]. In brief, the GAA enzyme was extracted overnight from the 3-mm DBS punch, and the aliquot was reacted with the enzyme substrate in the presence of acarbose at 37 °C for 20 h. The reaction was then terminated by the addition of 150 mM EDTA solution (pH 11.3–12.0). Finally, the accumulation of reaction products was determined using an ARVO fluorometer (PerkinElmer) (λex = 355 nm, λem = 460 nm).

## 3 Results

The LSD assay was validated in terms of (1) chromatographic separation of the enzyme reaction products using several commercially available reversed-phase columns, (2) QC validation using CDC-provided QC DBSs, (3) analysis of the enzyme activity in random neonates and LSD-confirmed individuals in a Japanese population, and (4) correlation of the activity of the GAA enzyme using LC-MS/MS and fluorometric methods.

### 3.1 Chromatographic separation

**Fig. 1:**
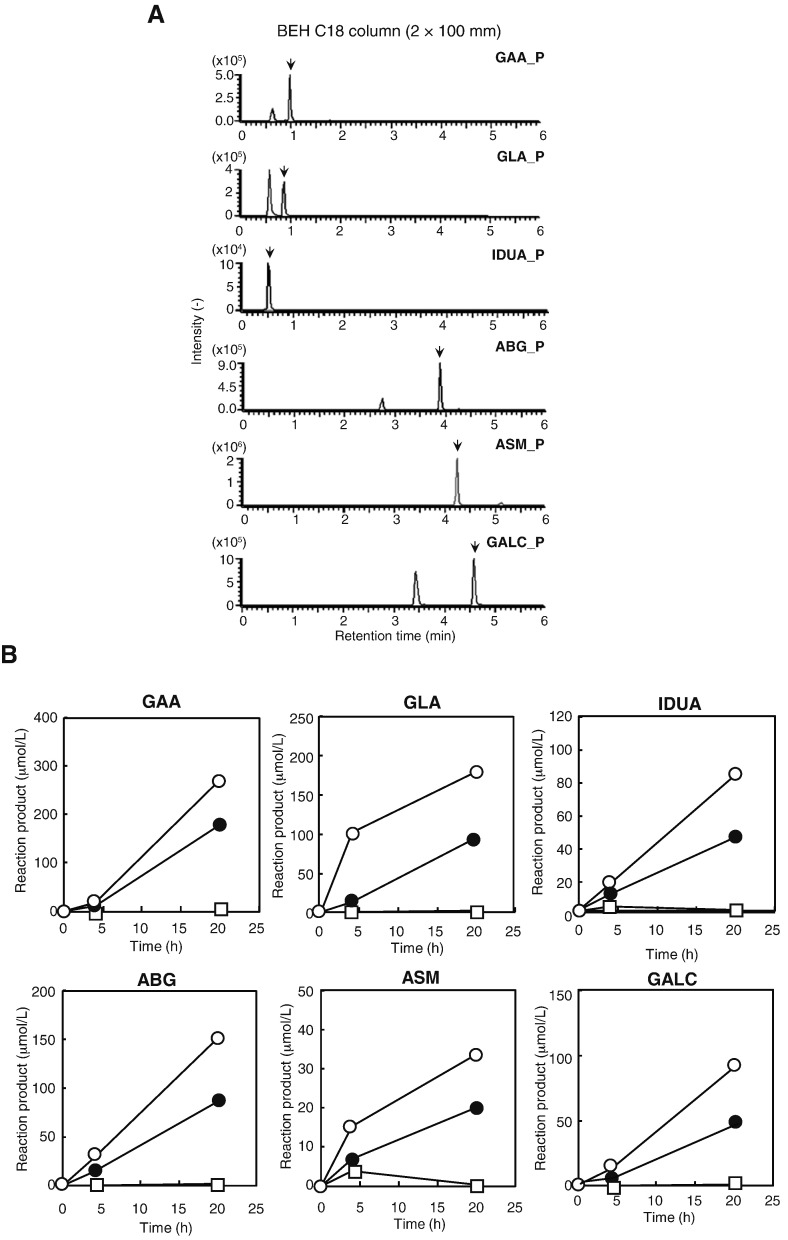
Formation of reaction products of the 6-plex enzyme assay. (A) Representative chromatograms of the reaction products of six LSD enzymes. A BEH C18 column (2 × 100 mm) was used. The arrows indicate the product of the enzyme reaction. P, product. (B) Time-dependent accumulation of enzyme reaction products of the LSD assay. The concentrations of the enzyme reaction products were determined at 0, 4 or 20 h of incubation at 37 °C. Details of the method are provided in the Experimental procedure section.

First, the chromatographic separation of the reaction products of the 6-plex LSD assay system was examined. Three commercially available analytical columns were tested: a silica-based conventional BEH C18 column (Waters) that is widely available worldwide and two silica-based monolith columns compatible with higher flow rates (a Chromolith RP-2 column [Merck-Millipore] and a MonoTower C18 column [GL Sciences]). Overall, the chromatographic properties of the three columns were almost the same. A BEH C18 column was used in a subsequent study of this assay due to its commercial availability, as the enzyme reaction products of GAA, GLA, IDUA, ABG, ASM, and GALC were readily detected under the analytical conditions tested (Fig. 1A). The peaks of the enzyme reaction products, indicated by arrows in Fig. 1A, were baseline separated from the corresponding in-source degraded compounds of the substrates of GAA, GLA, ABG, ASM, and GALC, leading to minimal inaccuracy in the measurement of the enzyme reaction products. The other two columns showed similar chromatographic behavior. The only difference in separation among the three columns was that the peak of substrate and internal standard for GALC migrated before those for ABG in the MonoTower C18 column (Supplementary Table 4). The accumulation of the enzyme reaction products of the six enzymes was almost linear over 20 h at 37 °C (Fig. 1B).

### 3.2 Validation of the assay using CDC-provided QC DBSs

**Fig. 2:**
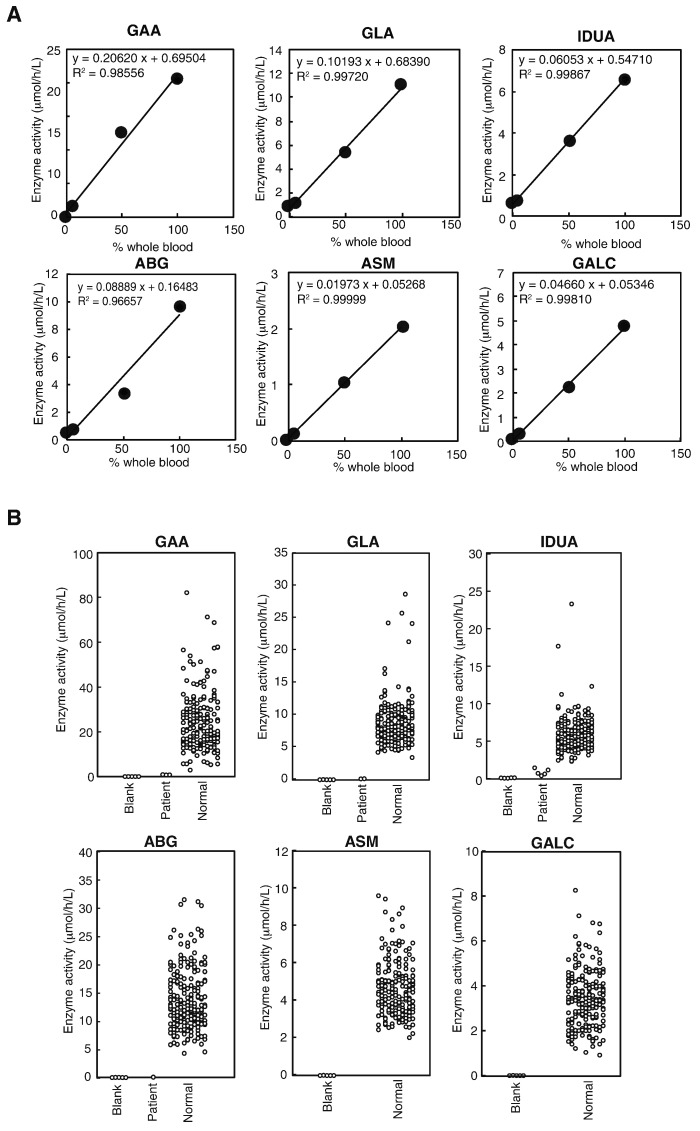
Enzyme activity of six LSDs in CDC-provided QC DBSs and clinical samples. (A) Calibration curves of the enzyme activities of the six LSD enzymes using CDC QC DBSs. The levels of the enzymes in the high, middle, low, and baseline QC DBSs were 100, 50, 5, and 0% of whole blood, respectively. The enzyme activities were determined after 20 h of incubation at 37 °C, as described in the Experimental Procedure section. (B) The levels of the activities of the six LSD enzymes in Japanese neonatal DBSs determined by LC-MS/MS (*n* = 210). The number of LSD-confirmed enzyme activity was as follows: GAA (*n* = 3), GLA (*n* = 2), IDUA (*n* = 5), and ABG (*n* = 1). Similarly, we analyzed enzyme activity for 6 LSDs in five filter papers for blank.

Second, the 6-plex LSD assay was validated using QC DBSs provided by the CDC [21]. For this purpose, the enzyme activities of the six LSDs were determined in high/middle/low/baseline QC DBSs containing 100, 50, 5, and 0% control enzyme activity, respectively. As shown in Fig. 2A, the linear correlation between the measured enzyme activity and nominal enzyme content in the QC DBSs was acceptable. Notably, we regularly obtained a good linear correlation (i.e. R^2^ > 0.95), which is within the acceptable level in clinical laboratory medicine. The intraday and interday coefficient of variation values for the high and middle CDC-provided QC DBSs were within 25% when assessed using the BEH C18 column (Table 1). On average, the activity of the enzymes in the CDC-provided QC DBSs by our measurement was 15.3 μmol/h/L for GAA (92% of the reported enzyme activity in CDC analytical information), 9.9 μmol/h/L for GLA (105%), 5.8 μmol/h/L for IDUA (43%), 8.4 μmol/h/L for ABG (74%), 1.7 μmol/h/L for ASM (56%), and 4.6 μmol/h/L for GALC (86%), respectively (*n* = 6). To ensure that the each measured enzyme activity derived from a single adult donor does not depend on the position in a 96-well plate, the enzyme activities in 24 different wells in the plate were examined. Overall, the difference in enzyme activities of the six LSDs from mean value was within 20% (Supplementary Fig. 1). The linearity of enzyme activity in the QC DBSs and intraday and interday coefficient variation values were also measured using the Chromolith column and found these were similar (data not shown; see Supplementary Table 5).

### 3.3 Examination of enzyme activities in clinical samples

Third, the enzyme activity of random neonates in a Japanese population was determined. As shown in Fig. 2B, the levels of enzyme activity for blank detected in filter paper samples were low. The detected enzyme activity in the blank samples was as follows: GAA, 0.011 ± 0.014 (min, 0.003; max, 0.037; median, 0.005); GLA, 0.010 ± 0.011 (min, 0.005; max, 0.029; median, 0.004); IDUA, 0.085 ± 0.028 (min, 0.053; max, 0.112; median, 0.090); ABG, 0.015 ± 0.018 (min, 0.003; max, 0.042; median, 0.004); ASM, 0.005 ± 0.006 (min, 0.001; max, 0.015; median, 0.002); and GALC, 0.005 ± 0.004 (min, 0.001; max, 0.012; median, 0.004) (mean ± SD, *n* = 5). The enzyme activity of random neonates in a Japanese population was as follows: GAA, 24.1 ± 12.5 (min, 2.9; max, 81.4; median, 21.3); GLA, 8.3 ± 3.3 (min, 3.3; max, 27.4; median, 7.7); IDUA, 5.6 ± 2.2 (min, 2.3; max, 22.8; median, 5.1); ABG, 13.0 ± 5.1 (min, 4.0; max, 29.5; median, 11.8); ASM, 4.5 ± 1.4 (min, 2.0; max, 9.3; median, 4.3); and GALC, 3.5 ± 1.4 (min, 0.9; max, 12.9; median, 3.4) (mean ± SD, *n* = 210) (Fig. 2B). The frequency distribution of the activity of each enzyme in this population was nearly bell shaped (Supplementary Fig. 2). The enzyme activities of GAA (*n* = 3), GLA (*n* = 2), IDUA (*n* = 5), and ABG (*n* = 1) in LSD-confirmed patients were lower than those of healthy controls.

### 3.4 Comparison of GAA enzyme activity determined by the LC-MS/MS and fluorescence methods

Finally, we compared the level of GAA enzyme activity determined by LC-MS/MS to that measured by the fluorescent method. The mean value of GAA enzyme activity in the population according to the fluorescence method was 17.0 ± 6.6 (mean ± SD, *n* = 210, min = 2.53, max = 46.1, median = 16.2 μmol/h/L), indicating that these values seemed similarly to those by LC-MS/MS (Table 2). We also found that a positive linear correlation between the measurements of the two methods (R = 0.3439).

## 4 Discussion

The measurement of the enzyme activities of the six aforementioned LSDs using MS/MS-based technology has become the gold standard in large-scale assays (Table 2) [7]. This technology uses MRM, which enables simultaneous detection of multiple compounds. Accurate quantitation of endogenously accumulated enzyme products can be achieved by the inclusion of internal standards, which have a similar, but not the same, chemical structure to that of enzyme reaction products with 5–7 deuterium. The substrates and internal standards used for the GAA and GLA assays in the present study were used as originally synthesized, whereas others have been re-designed [20]. A recent study reported that the assay using the new substrates yielded comparable results to those obtained using substrates employed previously (Table 2) [20]. The alteration of the chemical structure of the substrates and internal standards results in the co-migration of the formed products and internal standards in ABG, ASM, and GALC assays in a chromatographic run, thereby making quantitation of each enzyme activity for these three assays much more accurate, even in LC-MS/MS assays.

The superiority of the LC-MS/MS method as compared to the fluorometric method has been attributed to its analytical range, which is calculated by dividing the enzyme activity in the high QC sample by that in the filter paper for blank (Table 3) [23]. Recently, Elliott et al. reported that the analytical range of the 6-plex MS/MS assay was higher than that of various fluorometric methods [20]. In the present study, the analytical range of the LC-MS/MS method was much wider than that of the MS/MS method (Table 3, Table 4). This finding was expected, as the remaining impurities after liquid–liquid extraction, as well as substrate-derived degradation products, can be eliminated using HPLC before MS/MS detection (Fig. 1A). Undoubtedly, the MS/MS-based method offers higher throughput in studies of large numbers of specimens. Thus, all reported studies of large numbers of samples have used the MS/MS-based assay (Table 2). Given the wider analytical range of the LC-MS/MS-based method, it may be more practical for diagnostic applications.

The mean GAA activity reported in this study (*n* = 210) was higher than that previously reported (Table 2). Using the MS/MS method, a Korean study also reported that the mean GAA activity of them was approximately 24 μmol/h/L [24]. Thus, higher GAA activity may be limited to Asian populations. A previous study reported that GAA activity was stable at or below 4 °C under proper storage conditions [21]. Based on the current evidence, the average enzyme activity of the six LSD enzymes in a Japanese neonatal population reported in the present study may be considered preliminary estimates.

In conclusion, the present study examined the applicability of the 6-plex LSD assay using LC-MS/MS to neonatal screening. As enzyme activity depends on the population, region, and country, the cut-off value for neonatal screening needs to be based on assays of samples derived from the local population. The wider analytical range of the LC-MS/MS method compared to the fluorometric method clearly distinguishes the LSD-affected individuals from healthy controls.

## Figures and Tables

**Table 1:** Intraday and interday assay precision for multiple analyses of the CDC quality control samples.^a^

CDC QC	Intraday CV (%)						Interday CV (%)					
	GAA	GLA	IDUA	ABG	ASM	GALC	GAA	GLA	IDUA	ABG	ASM	GALC
High (100% whole blood)	2.5	12.7	5.1	3.3	2.0	2.4	15.4	11.4	13.5	23.3	16.9	8.7
Middle (50% whole blood)	4.1	0.9	5.0	8.7	1.6	8.5	12.1	7.6	13.5	16.3	9.0	20.4
Low (5% whole blood)	8.0	23.7	21.8	13.5	4.4	12.5	16.4	9.7	41.9	20.4	24.1	7.9
Base (0% whole blood)	15.8	12.2	14.0	12.6	10.7	14.8	68.9	8.7	38.4	19.2	54.6	19.8
Replicate (*n*)	5	5	5	5	5	5	5	5	5	5	5	5

a Data obtained using a BEH C18 column.

**Table 2:** Comparison of LSD enzyme activities determined by mass spectrometry-based assay.

Investigator	Mashima R	Cho SE	Gucciardi A	Mechtler TP	Metz TF	Elliott S	Liao HC	Scott CR	Wittmann J	Orsini JJ	Dajnoki A
Year	2016	2016	2014	2012	2011	2016	2014	2013	2012	2012	2008
Country/area	Japan	Korea	Italy	Austria	Austria	WA	Taiwan	WA	Hungary	US	Austria
Method	LC-MS/MS	LC-MS/MS	LC-MS/MS	LC-MS/MS	LC-MS/MS	MS/MS	MS/MS	MS/MS	MS/MS	MS/MS	MS/MS
Substrate	New	Old	Old	Old	Old	New	Old	Old	Old	Old	Old
*n*	210	1526–1606	1136	825	8586	42,391–44,485	103–191 k	106–111 k	40,024	5055	10,279

Enzyme activity^a^											
GAA	24.1	17.7	20.5	18.6	22.5	12.4	16.7	17.6	15.0	16.8	14.7
GLA	8.3	8.5	14.1	6.4	10	17.3	6.7	10.2	11.0	20.7	ND
IDUA	5.6	ND	12.2	12.4	7.9	6.6	ND	3.6	ND	ND	ND
ABG	13.0	26.8	14.3	19.6	16.8	12.7	22.6	ND	17.7	15.1	ND
ASM	4.5	ND	0.3	4.4	8.9	6.0	ND	ND	9.2	22.2	ND
GALC	3.5	ND	1.49	ND	1.27	5.0	ND	ND	ND	3.6	ND
Reference	This study	[25]	[26]	[27]	[28]	[20]	[19]	[18]	[17]	[29]	[30]

*n*, number of individuals; ND, not determined. WA, Washington state.

a μmol/h/L.

**Table 3:** The analytical range for 6 LSDs using LC-MS/MS.

	GAA	GLA	IDUA	ABG	ASM	GALC
QC high (μmol/h/L blood)	20.5	11.1	6.6	9.6	2.0	4.8
Filter paper blank (μmol/h/L blood)	0.01	0.01	0.10	0.02	0.01	0.01
Analytical range^a^	1554	1007	65	515	370	872

a Analytical range is defined by the enzyme activity in QC High DBS divided by that in filter paper blank as reported previously [20].

**Table 4:** Comparison of analytical ranges for GAA activity determined by different methods.

Method	Analytical range	Reference
HPLC-MS/MS	1554	This study
MS/MS (Washington state)	88	[20]
MS/MS (New York state)	66	[20]
4MU	16.6	[23]
4MU	4.9	This study

## Appendix A Supplementary data

Supplementary Tables 1-4, Instrumentation of UHPLC-MS/MS used in this study; Supplementary Table 5, Intraday and interday assay CV values obtained by a Chromolith column; Supplementary Figure 1, Assay validation using a 96-well plate; Supplementary Figure 2, Frequency distribution of enzyme activities in a Japanese population. Image 1

## References

[1] Platt F.M., Boland B., van der Spoel A.C. (2012). The cell biology of disease: lysosomal storage disorders: the cellular impact of lysosomal dysfunction. J. Cell Biol..

[2] Boustany R.M. (2013). Lysosomal storage diseases–the horizon expands. Nat. Rev. Neurol..

[3] Escolar M.L., Poe M.D., Provenzale J.M., Richards K.C., Allison J., Wood S., Wenger D.A., Pietryga D., Wall D., Champagne M., Morse R., Krivit W., Kurtzberg J. (2005). Transplantation of umbilical-cord blood in babies with infantile Krabbe's disease. N. Engl. J. Med..

[4] Cheng S.H. (2014). Gene therapy for the neurological manifestations in lysosomal storage disorders. J. Lipid Res..

[5] Platt F.M. (2014). Sphingolipid lysosomal storage disorders. Nature.

[6] Lukina E., Watman N., Arreguin E.A., Banikazemi M., Dragosky M., Iastrebner M., Rosenbaum H., Phillips M., Pastores G.M., Rosenthal D.I., Kaper M., Singh T., Puga A.C., Bonate P.L., Peterschmitt M.J. (2010). A phase 2 study of eliglustat tartrate (Genz-112638), an oral substrate reduction therapy for Gaucher disease type 1. Blood.

[7] Gelb M.H., Scott C.R., Turecek F. (2015). Newborn screening for lysosomal storage diseases. Clin. Chem..

[8] Levesque S., Auray-Blais C., Gravel E., Boutin M., Dempsey-Nunez L., Jacques P.E., Chenier S., Larue S., Rioux M.F., Al-Hertani W., Nadeau A., Mathieu J., Maranda B., Desilets V., Waters P.J., Keutzer J., Austin S., Kishnani P. (2016). Diagnosis of late-onset Pompe disease and other muscle disorders by next-generation sequencing. Orphanet J. Rare Dis..

[9] Desnick R.J., Brady R., Barranger J., Collins A.J., Germain D.P., Goldman M., Grabowski G., Packman S., Wilcox W.R. (2003). Fabry disease, an under-recognized multisystemic disorder: expert recommendations for diagnosis, management, and enzyme replacement therapy. Ann. Intern. Med..

[10] Muenzer J., Wraith J.E., Clarke L.A. (2009). Mucopolysaccharidosis I: management and treatment guidelines. Pediatrics.

[11] Grabowski G.A., Zimran A., Ida H. (2015). Gaucher disease types 1 and 3: phenotypic characterization of large populations from the ICGG Gaucher registry. Am. J. Hematol..

[12] Schuchman E.H. (1895–1900). Acid Sphingomyelinase, Cell Membranes and Human Disease: Lessons from Niemann-Pick Disease FEBS Lett 584 (2010).

[13] Chamoles N.A., Blanco M.B., Gaggioli D., Casentini C. (2001). Hurler-like phenotype: enzymatic diagnosis in dried blood spots on filter paper. Clin. Chem..

[14] Hwu W.L., Chien Y.H., Lee N.C., Chiang S.C., Dobrovolny R., Huang A.C., Yeh H.Y., Chao M.C., Lin S.J., Kitagawa T., Desnick R.J., Hsu L.W. (2009). Newborn screening for Fabry disease in Taiwan reveals a high incidence of the later-onset GLA mutation c.936 + 919G > A (IVS4 + 919G > A). Hum. Mutat..

[15] Oda E., Tanaka T., Migita O., Kosuga M., Fukushi M., Okumiya T., Osawa M., Okuyama T. (2011). Newborn screening for Pompe disease in Japan. Mol. Genet. Metab..

[16] Mechtler T.P., Stary S., Metz T.F., De Jesus V.R., Greber-Platzer S., Pollak A., Herkner K.R., Streubel B., Kasper D.C. (2012). Neonatal screening for lysosomal storage disorders: feasibility and incidence from a nationwide study in Austria. Lancet.

[17] Wittmann J., Karg E., Turi S., Legnini E., Wittmann G., Giese A.K., Lukas J., Golnitz U., Klingenhager M., Bodamer O., Muhl A., Rolfs A. (2012). Newborn screening for lysosomal storage disorders in Hungary. JIMD Rep..

[18] Scott C.R., Elliott S., Buroker N., Thomas L.I., Keutzer J., Glass M., Gelb M.H., Turecek F. (2013). Identification of infants at risk for developing Fabry, Pompe, or mucopolysaccharidosis-I from newborn blood spots by tandem mass spectrometry. J. Pediatr..

[19] Liao H.C., Chiang C.C., Niu D.M., Wang C.H., Kao S.M., Tsai F.J., Huang Y.H., Liu H.C., Huang C.K., Gao H.J., Yang C.F., Chan M.J., Lin W.D., Chen Y.J. (2014). Detecting multiple lysosomal storage diseases by tandem mass spectrometry–a national newborn screening program in Taiwan. Clin. Chim. Acta.

[20] Elliott S., Buroker N., Cournoyer J.J., Potier A.M., Trometer J.D., Elbin C., Schermer M.J., Kantola J., Boyce A., Turecek F., Gelb M.H., Scott C.R. (2016). Pilot study of newborn screening for six lysosomal storage diseases using tandem mass spectrometry. Mol. Genet. Metab..

[21] De Jesus V.R., Zhang X.K., Keutzer J., Bodamer O.A., Muhl A., Orsini J.J., Caggana M., Vogt R.F., Hannon W.H. (2009). Development and evaluation of quality control dried blood spot materials in newborn screening for lysosomal storage disorders. Clin. Chem..

[22] Spacil Z., Tatipaka H., Barcenas M., Scott C.R., Turecek F., Gelb M.H. (2013). High-throughput assay of 9 lysosomal enzymes for newborn screening. Clin. Chem..

[23] Kumar A.B., Masi S., Ghomashchi F., Chennamaneni N.K., Ito M., Scott C.R., Turecek F., Gelb M.H., Spacil Z. (2015). Tandem mass spectrometry has a larger analytical range than fluorescence assays of lysosomal enzymes: application to newborn screening and diagnosis of mucopolysaccharidoses types II, IVA, and VI. Clin. Chem..

[24] Han M., Jun S.H., Song S.H., Park K.U., Kim J.Q., Song J. (2011). Use of tandem mass spectrometry for newborn screening of 6 lysosomal storage disorders in a Korean population Korean. J. Lab. Med..

[25] Cho S.E., Kwak J.R., Lee H., Seo D.H., Song J. (2016). Triplex tandem mass spectrometry assays for the screening of 3 lysosomal storage disorders in a Korean population. Clin. Chim. Acta.

[26] Gucciardi A., Legnini E., Di Gangi I.M., Corbetta C., Tomanin R., Scarpa M., Giordano G. (2014). A column-switching HPLC-MS/MS method for mucopolysaccharidosis type I analysis in a multiplex assay for the simultaneous newborn screening of six lysosomal storage disorders. Biomed. Chromatogr..

[27] Mechtler T.P., Metz T.F., Muller H.G., Ostermann K., Ratschmann R., De Jesus V.R., Shushan B., Di Bussolo J.M., Herman J.L., Herkner K.R., Kasper D.C. (2012). Short-incubation mass spectrometry assay for lysosomal storage disorders in newborn and high-risk population screening. J. Chromatogr. B Analyt. Technol. Biomed. Life Sci..

[28] Metz T.F., Mechtler T.P., Orsini J.J., Martin M., Shushan B., Herman J.L., Ratschmann R., Item C.B., Streubel B., Herkner K.R., Kasper D.C. (2011). Simplified newborn screening protocol for lysosomal storage disorders. Clin. Chem..

[29] Orsini J.J., Martin M.M., Showers A.L., Bodamer O.A., Zhang X.K., Gelb M.H., Caggana M. (2012). Lysosomal storage disorder 4 + 1 multiplex assay for newborn screening using tandem mass spectrometry: application to a small-scale population study for five lysosomal storage disorders. Clin. Chim. Acta.

[30] Dajnoki A., Muhl A., Fekete G., Keutzer J., Orsini J., Dejesus V., Zhang X.K., Bodamer O.A. (2008). Newborn screening for Pompe disease by measuring acid alpha-glucosidase activity using tandem mass spectrometry. Clin. Chem..

