# Supplementary material for: Levels of enzyme activities in six lysosomal storage diseases in Japanese neonates determined by liquid chromatography-tandem mass spectrometry
Source: Mol Genet Metab Rep. 2016 Aug 31;9:6–11. doi: 10.1016/j.ymgmr.2016.08.007 (PMC5011175; doi:10.1016/j.ymgmr.2016.08.007)
Supplement: Supplementary file 1 — Supplementary Tables 1-4, Instrumentation of UHPLC-MS/MS used in this study; Supplementary Table 5, Intraday and interday assay CV values obtained by a Chromolith column; Supplementary Figure 1, Assay validation using a 96-well plate; Supplementary Figure 2, Frequency distribution of enzyme activities in a Japanese population. [file mmc1.pdf]

Supplementary Table 1

UPLC method for the measurement of enzyme activities for 6 LSDs.

Column temperature	40°C
Weak wash solvent	MeOH/CH_3CN/IPA/0.1%aqueous formic acid
Strong wash solvent	MeOH/CH_3CN/IPA/0.1%aqueous formic acid
Mobile phase	CH_3CN/H_2O/0.2% formic acid in H_2O
Flow rate	0.3 mL/min
Injection volume	10 μL
Injection mode	Direct injection
Autosampler temperature	4°C

LSD, lysosomal storage disorder.

Supplementary Table 2

Instrument parameters for the measurement of substrates, internal standards and products of 6 LSD enzyme activities using LC-MS/MS.

Interface	ESI
Polarity	Positive
Capillary voltage	2.0 kV
Source temperature	120°C
Desolvation temperature	450°C
Flow rate of nebulizing gas	800L/h
Flow rate of drying gas	50 L/h
Analyzing mode	MRM
Scan time	0.2 s
Data format	Centroid

Supplementary Table 3

Product ion, precursor ion, cone voltage and collision energy for the fragmentation of substrates, products and internal standards using LC-MS/MS.

Species	Precursor ion (*m/z*)	Product ion (*m/z*)	Cone (V)	Collision energy (V)
GAA-S	660.4	560.3	25	22
GAA-P	498.3	398.3	10	20
GAA-IS	503.4	403.3	10	16
GLA-S	646.4	546.3	23	19
GLA-P	484.3	384.2	22	12
GLA-IS	489.3	389.3	22	12
IDUA-S	602.3	317.2	35	14
IDUA-P	426.2	317.2	20	12
IDUA-IS	431.3	322.2	20	15
ABG-S	546.4	264.2	20	15
ABG-P	384.4	264.3	15	12
ABG-IS	391.4	271.3	15	12
ASM-S	563.4	184.0	15	30
ASM-P	398.4	264.3	15	20
ASM-IS	405.4	264.3	15	20
GALC-S	574.5	264.3	20	25
GALC-P	412.4	264.3	20	15
GALC-IS	417.4	264.3	20	18

GAA, α-glucosidase; GLA, α-galactosidase A ; IDUA, α-L-iduronidase; ABG, glucocerebrosidase; ASM, acid sphingomyelinase; GALC, galactosylceramidase.

Supplementary Table 4

Retention times for the substrates, products and internal standards of assay for lysosomal enzyme activities using LC-MS/MS.

Column	Column 1 (ACQUITY BEH C18)	Column 2 (Chromolith)	Column 3 (Mono Tower C18)
Flow rate (mL/min)	0.3	0.3	0.6
Species	Retention time (min)		
GAA-S	0.63	0.74	1.16
GAA-P	0.99	1.04	1.59
GAA-IS	0.98	1.04	1.59
GLA-S	0.57	0.70	1.10
GLA-P	0.85	0.94	1.48
GLA-IS	0.86	0.94	1.48
IDUA-S	0.50	0.63	0.95
IDUA-P	0.53	0.66	1.07
IDUA-IS	0.53	0.66	1.05
ABG-S	2.73	2.36	2.81
ABG-P	3.85	3.30	3.71
ABG-IS	3.85	3.30	3.70
ASM-S	2.49	2.40	2.61
ASM-P	4.21	3.58	2.09
ASM-IS	4.20	3.58	2.10
GALC-S	3.42	2.89	3.27
GALC-P	4.56	3.84	2.80
GALC-IS	4.56	3.84	2.78

Supplementary Table 5

Intraday and interday assay precision for multiple analyses of the CDC quality control samples.[a]

	Intraday CV (%)						Interday CV (%)					
CDC QC	GAA	GLA	IDUA	ABG	ASM	GALC	GAA	GLA	IDUA	ABG	ASM	GALC
High (100% whole blood)	12.1	8.3	12.5	0.6	8.9	6.8	7.4	5.5	14.1	18.0	17.3	10.5
Middle (50% whole blood)	18.2	9.6	16.5	10.1	2.9	8.9	11.9	4.0	19.8	22.1	11.1	14.7
Low (5% whole blood)	25.8	15.7	4.0	5.7	12.9	10.1	10.9	27.0	47.0	17.8	6.7	2.9
Base (0% whole blood)	52.9	36.5	18.9	9.5	12.0	7.3	17.1	38.1	56.0	19.0	4.6	7.6
Replicate (*n*)	3	3	3	3	3	3	4	4	4	4	4	4

[a]Data obtained using a Chromolith column.

Figure

A

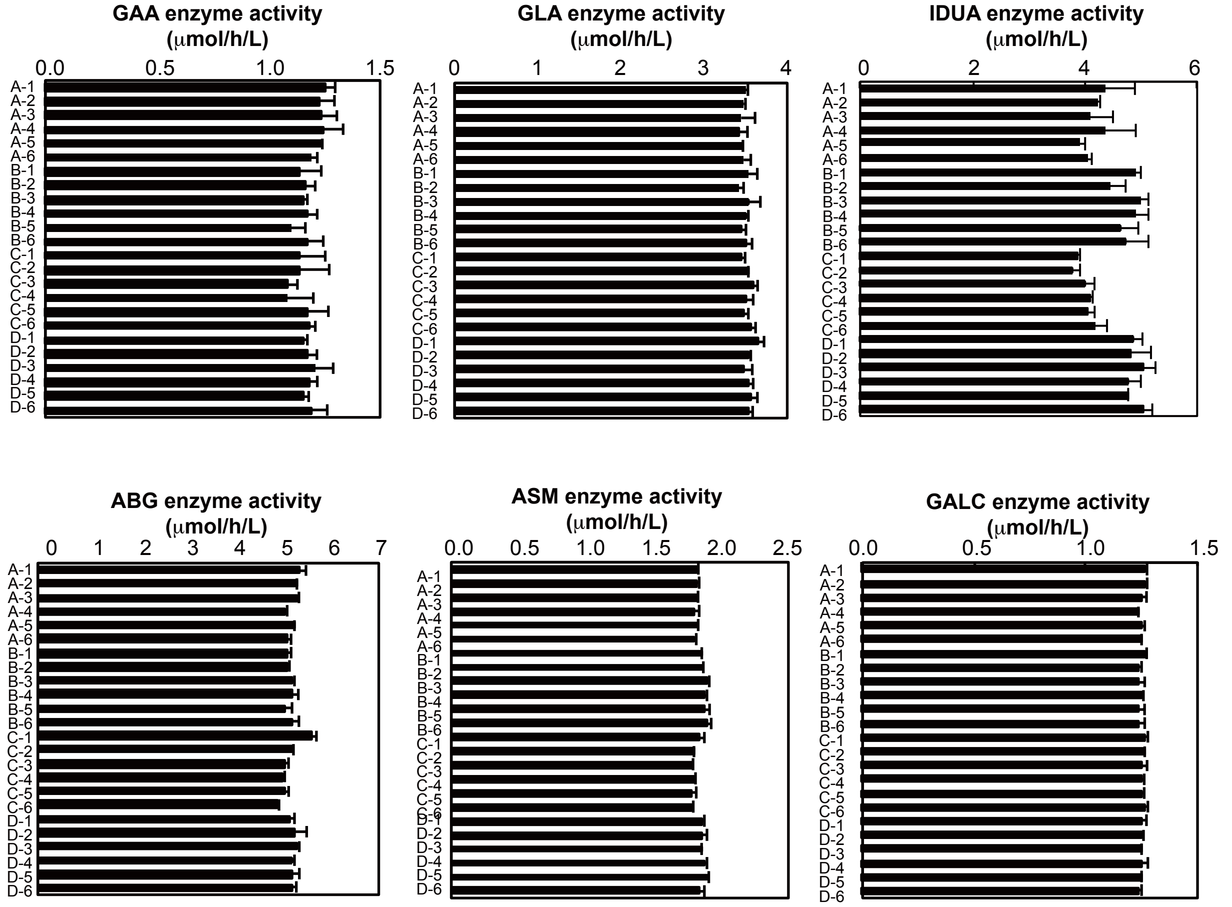

B

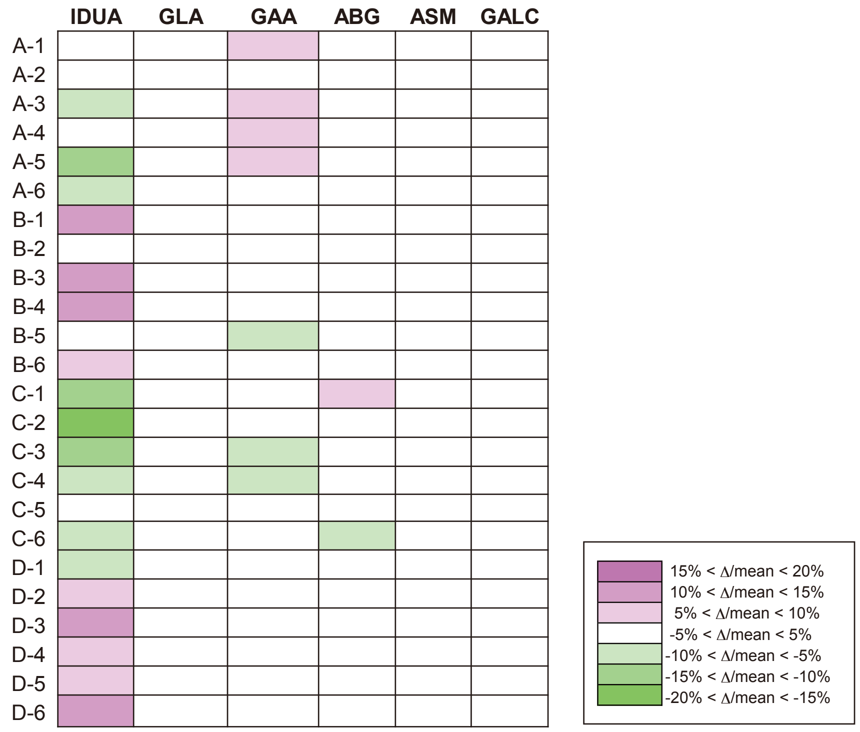

Suppelmentary Figure 1
Mashima R et al

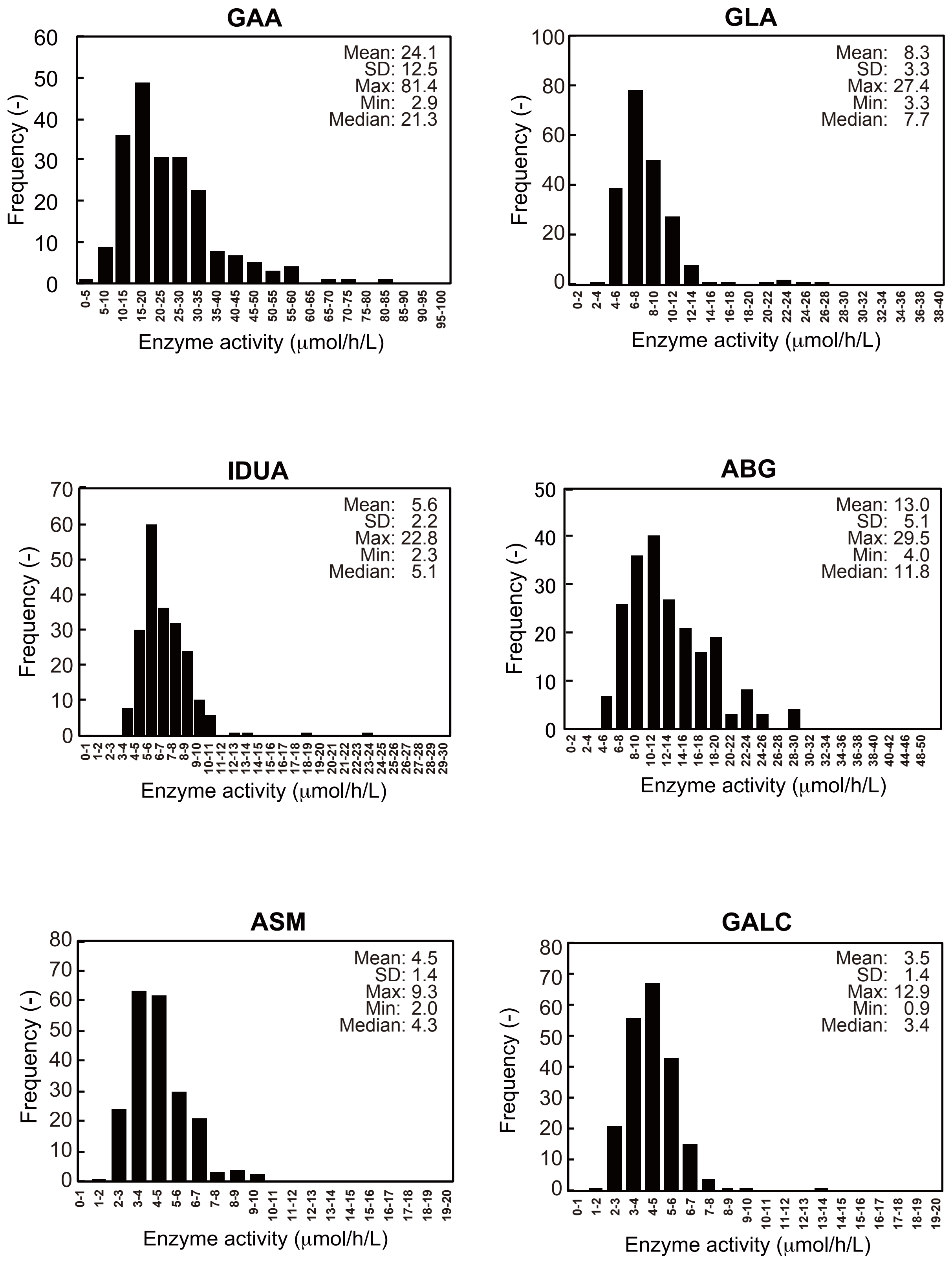

Supplementary Figure 2

Mashima R et al
